# A Clinical Decision Support System for Femoral Peripheral Arterial Disease Treatment

**DOI:** 10.1155/2013/898041

**Published:** 2013-12-08

**Authors:** Alkın Yurtkuran, Mustafa Tok, Erdal Emel

**Affiliations:** ^1^Department of Industrial Engineering, Faculty of Engineering, Görükle Campus, Uludag University, 16059 Bursa, Turkey; ^2^Department of Thoracic and Cardiovascular Surgery, Faculty of Medicine, Görükle Campus, Uludag University, 16059 Bursa, Turkey

## Abstract

One of the major challenges of providing reliable healthcare services is to diagnose and treat diseases in an accurate and timely manner. Recently, many researchers have successfully used artificial neural networks as a diagnostic assessment tool. In this study, the validation of such an assessment tool has been developed for treatment of the femoral peripheral arterial disease using a radial basis function neural network (RBFNN). A data set for training the RBFNN has been prepared by analyzing records of patients who had been treated by the thoracic and cardiovascular surgery clinic of a university hospital. The data set includes 186 patient records having 16 characteristic features associated with a binary treatment decision, namely, being a medical or a surgical one. K-means clustering algorithm has been used to determine the parameters of radial basis functions and the number of hidden nodes of the RBFNN is determined experimentally. For performance evaluation, the proposed RBFNN was compared to three different multilayer perceptron models having Pareto optimal hidden layer combinations using various performance indicators. Results of comparison indicate that the RBFNN can be used as an effective assessment tool for femoral peripheral arterial disease treatment.

## 1. Introduction

Various engineering techniques have been adapted to health care delivery systems and the quality of health care services has been improved using these artificial intelligence techniques. It has been proven that introducing machine learning tools into clinical decision support systems can easily increase the decision accuracy and decrease costs and the dependency on highly qualified specialists. Since artificial neural networks (ANN) can easily be trained for identifying the patterns and extracting rules using a small number of cases, they are widely used as a powerful tool for clinical decision support systems [1].

Peripheral arterial disease (PAD) is a common pathologic disease worldwide. Peripheral arterial disease is a disease in which plaque, which is made up of fat, cholesterol, calcium, fibrous tissue, and other substances in the blood, builds up in the arteries that carry blood to head, organs, and limbs. PAD affects more than 30 million people worldwide, and while it can strike anyone, it is most common in people over age 65 [2].

PAD is associated with a significant burden in terms of morbidity and mortality, due to claudication, rest pain, ulcerations, and amputations. In case of mild or moderate peripheral arterial diseases, a medical or conservative therapy can be chosen but the gold-standard treatment of severe PAD is a surgical or an endovascular revascularization [2]. However, up to 30% of patients are not candidates for such interventions, due to excessive surgical risks or unfavorable vascular involvements. The presence of diffuses and multiple and distal arterial stenosis renders successful revascularization sometimes impossible. These “no-option” patients are left to medical therapy, which may slow the progression of disease at best [3].

It is very difficult to decide whether surgical or medical treatment is the best option since PAD depends on many factors like anatomic location, symptoms, comorbidities, and risk about cardiac condition or anesthesia. Cardiovascular surgeons should prefer the best appropriate choice of treatment and most of the time the decision allows the surgeon with his own experience. Cardiovascular specialists widely use intersociety Consensus for the classification of PADs' (TASC II) (Trans-Atlantic intersociety Consensus), which is based on the anatomic locations of lesions [3].

In this work, we present a clinical treatment decision support system using a radial basis function neural network (RBFNN) in order to help doctors to make an accurate treatment decision for patients having femoral PAD. Proposed RBFNN was compared to three different multilayer perceptron (MLP) networks and results indicate that the proposed RBFNN outperforms MLP networks. Based on our extensive literature review, no previous study was carried out which included a decision support system for clinical treatment of femoral PAD.

The remainder of this paper is organized as follows.  Section 2 summarizes previous studies; Section 3 covers the clinical data and input and output features of the proposed model.  Section 4 gives a brief introduction to the RBFNN and experiments. Related results are given in Section 5 and finally Section 6 concludes the paper.

## 2. Related Work

In recent years, there have been many studies that focused on decision support systems to improve the accuracy of decisions for diagnosis and treatment of diseases. Such decision support systems frequently depend on ANN-based perceptive algorithms that are built upon previous patient records.

To cite a few but significant works of others, Mehrabi et al. [4] used a MLP network and a RBFNN to classify chronic obstructive pulmonary (COPD) and congestive heart failure (CHF) diseases. They used Bayesian regularization to enhance the performance of MLP network. Moreover, they integrated K-means clustering algorithm and k-nearest neighborhood, to define centers for hidden neurons and to identify the spread, respectively. They have shown that both COPD and CHF have been classified using the MLP networks and the RBFNN accurately.

Subashini et al. [5] proposed a polynomial kernel for the support vector machine (SVM) and the RBFNN for ascertaining the diagnostic accuracy of cytological data obtained from the Wisconsin breast cancer database. They have shown that RBFNN outperformed SVM for accurately classifying the tumors. Lewenstein [6] used RBFNN as a tool for diagnosis of coronary artery disease. The research was performed using 776 data records and over 90% accuracy was achieved for classifying.

A short review of recent studies reveal numerous use of ANN techniques for diagnosis of diabetes mellitus [7–12], chest diseases [13–17], Parkinson disease [18, 19], breast cancer [5, 20–23], thyroid disease [24–26] and cardiovascular diseases [4, 6, 27–36].

Broomhead and Lowe [37] were the first to use the RBFNN in designing neural networks. In recent years, the RBFNN have attracted extensive research interest. [38–42] Wu et al. [19] used RBFNN to accurately identify Parkinson's disease. The data for training the RBFNN was obtained by means of deep brain electrodes implanted into a Parkinson's disease patient's brain. The output of the study indicated that RBFNNs could be successfully designed and used to identify tremors on set pattern even for small number of spikes.

## 3. The Clinical Data

The input data set for training ANNs has been obtained from discharge reports dated from 2008 to 2012 within patient records of the department of thoracic and cardiovascular surgery clinic of a university hospital. 186 records with 114 male patients aged around 53 ± 7 and with 72 female patients aged as 58 ± 5 have been analyzed. Each patient's report contains one final treatment decision, which is taken here as an output class value of the corresponding input data set that is as follows.

- Class 1: medical treatment decision (89 patients).
- Class 2: surgery or endovascular treatment decision (97 patients).

All samples have a total of 16 features and these features were determined by consultations with cardiologists, surgeons, and anesthetists. Features, output classes and their normalized values are given in Table 1. Description of selected features is summarized in Tables 2–5.

## 4. Radial Basis Function Neural Network (RBFNN)

**Figure 1:**
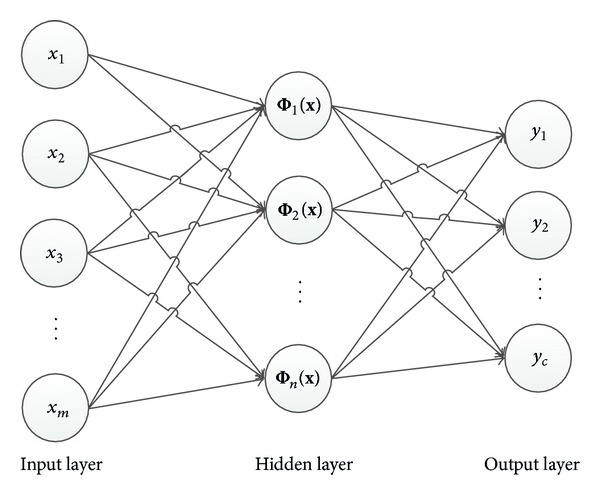
An example of RBFNN.

The RBFNN [43] has a feed forward architecture with 3 layers: (i) an input layer, (ii) a hidden layer, and (iii) an output layer. A typical RBFNN is shown in Figure 1. The input layer of *m* nodes accepts *m*-dimensional features as input data vector. The hidden layer, which is fully connected to the input layer, is composed of *n* radial basis function neurons. Each hidden layer neuron operates as a radial basis function that does a nonlinear mapping of feature space into output space. The output layer consists of *c* neurons, which calculate the weighted sum of the output of the each hidden layer node.

The most commonly employed radial basis function for hidden layers is the Gaussian function [44, 45] and is determined by mean vectors (cluster centers) ***μ***_*j*_ and covariance matrices **C**_*j*_ where *j* = 1,…, *n*. Covariance matrices are assumed to be in the form **C**_*j*_ = *σ*_*j*_^2^ **I**.

Let Φ_*j*_(**x**) be the Gaussian function representing the *j*th hidden node defined as

(1) $$\begin{array}{c} \Phi_{j}\left(x\right)=\exp\left(-\frac{{||x-\mu_{j}||}^{2}}{2\sigma_{j}^{2}}\right), \end{array}$$

where **x** = [*x*_1_,*x*_2_,…,*x*_*m*_]^*T*^ is the input feature vector, ***μ***_*j*_ = [*μ*_1*j*_,*μ*_2*j*_,…,*μ*_*mj*_]^*T*^ and *σ*_*j*_^2^ are the mean vector and the variance of the *j*th neuron, respectively. The *k*th output of the RBFNN is computed according to (2)

(2) $$\begin{array}{c} y_{k}=\sum_{j=1}^{n}w_{jk}\Phi_{j}\left(x\right)+\;w_{0k}. \end{array}$$

In (2), **w**_*k*_ = [*w*_1*k*_, *w*_2*k*_,…, *w*_*nk*_] is the vector of the weights between hidden and output layer and *w*_0*k*_ is the bias for *k* = 1,…, *c*. In order to design a RBFNN, the value of mean vectors (***μ***_*j*_) representing the location of cluster centers and variances (*σ*_*j*_^2^) for hidden neurons have to be calculated first. K-means clustering algorithm is used to determine the value of mean vectors which is given as follows.

**Step 1**

Initialize by choosing *m* random values for *n* hidden nodes (*μ*_*ij*_, *i* = 1,…, *m*, *j* = 1,…, *n*) as initial cluster centers.

**Step 2**

Assign a randomly selected input data sample **x** to the nearest *j*th cluster center using the Euclidean norm.

**Step 3**

Recalculate ***μ***_*j*_ including the assigned sample.

**Step 4**

Repeat Steps 2 and 3 until mean vectors do not change (***μ***_*j*_^new^≅***μ***_*j*_^old^).

The number of hidden neurons *n*, which should be determined experimentally, is effective on the performance of the RBFNN. Generally, it is assumed that variances of all clusters are identical and equal to *σ* ^2^ which is calculated as follows

(3) $$\begin{array}{c} \sigma^{2}=\frac{\eta d^{2}}{2}, \end{array}$$

where *d* is the maximum distance between cluster centers and *η* is an empirical scale factor and controls the smoothness of the nonlinear mapping function. Once the location of centers and their variances are determined, weights between the hidden layer and the output layer can be calculated. Equation (2) may be rewritten in the vector form as

(4) $$\begin{array}{c} Y=H\cdot W. \end{array}$$

In (4), **Y** is the (*n* × 1) dimensional output vector, **H** is the (*n* × (*m* + 1)) dimensional hidden neuron matrix, and **W** is the ((*m* + 1) × 1) dimensional weight vector. To reduce the computational effort **W** is directly calculated from the least squares pseudoinverse by (5)

(5) $$\begin{array}{c} W={\left(H^{T}H\right)}^{-1}H^{T}Y. \end{array}$$

## 5. Experiments

### 5.1. Measures for Performance Evaluation

In our experiments, in order to evaluate the performance of the proposed RBFNN effectively and accurately, several performance indicators such as area under the receiving operating characteristics curve (AUC), *accuracy*, *sensitivity* (*recall*), *specificity*, *positive predictive value* (PPV) (*precision)*, *negative predictive value* (NPV), *F-score*, and *Yuden Index* are analyzed [35, 36]. All these performance indicators are determined by using a confusion matrix, which is composed of the results of a binary (true/false) classification in terms of true positive (tp), false positive (fp), false negative (fn), and true negative (tn) counts. A confusion matrix for a binary classification is presented in Table 6. *Accuracy* is used to assess the overall effectiveness of the classifier (see (6)). *Sensitivity* is the ratio of correctly classified samples to all samples in that class (see (7)). *Specificity* measures the proportion of negatives, which are correctly identified (see (8)). PPV is the accuracy in a specified class (see (9)) and NPV is the proportion of cases with negative results that are correctly classified (see (10)). Finally, *F-measure* and *Yuden Index*, which are widely used performance indicators to assess neural network classification performances, are depicted in (11) and (12). Another important performance indicator of neural networks is the area under the receiving operating characteristics curve (AUC). Receiving operating characteristics curve is constructed by plotting the *sensitivity* versus (*1-specificity*) values for variety of cutoff points between 0.00 and 1.00. Furthermore, the *Hosmer-Lemeshow* (*H-L*) chi-square statistic is used as a numerical indicator of overall calibration

(6) $$\begin{array}{c} \text{Accuracy}=\frac{\text{tp}+\text{tn}}{\text{tp}+\text{fp}+\text{fn}+\text{tn}}, \end{array}$$

(7) $$\begin{array}{c} \text{Sensitivity}=\frac{\text{tp}}{\text{tp}+\text{fn}}, \end{array}$$

(8) $$\begin{array}{c} \text{Specificity}=\frac{\text{tn}}{\text{tn}+\text{fp}}, \end{array}$$

(9) $$\begin{array}{c} \text{PPV}=\frac{\text{tp}}{\text{fp}+\text{tp}}, \end{array}$$

(10) $$\begin{array}{c} \text{NPV}=\frac{\text{tn}}{\text{tn}+\text{fn}}, \end{array}$$

(11) $$\begin{array}{c} F_{\text{score}}=\frac{2\times \text{Sensitivity}\times \text{PPV}}{\text{Sensitivity}+\text{PPV}}, \end{array}$$

(12) $$\begin{array}{c} \text{Yuden}\;\text{Index}=\text{sensitivity}+\text{specificity}-1. \end{array}$$

### 5.2. Computational Results

Neural networks are prone to overfitting, especially when there are only a limited number of data. In order to estimate the performance of the neural networks accurately by reducing the bias and the variance on predicted results, 10-fold cross-validation method is used in this study. Multifold cross-validation, in which dynamic sets of validation and test data are used, is an efficient technique to avoid overfitting compared to regularization, early stopping, or data pruning especially when data are very scarce [43]. In 10-fold cross-validation, a data set is randomly partitioned into 10 equal subsamples having approximately equal number of samples from each class. Using this data set, while the RBFNN training is done by the first nine subsamples, the validation is done only by the last subsample. This training and testing process is repeated for 10 times by rotating each subsample to be used only once as the validation subsample. The mean and standard deviation of performance indicators for each neural network model are then reported.

In this study, as mentioned in Section 4, the cluster center locations for all Gaussian functions, which are employed as radial basis functions, are determined using K-means clustering algorithm. Network weights of the output layer are determined by the pseudoinverse method (4). Following preliminary tests, the empirical scale factor is set to *η* = 0.6. For simplicity and ease of calculation, it is assumed that all variances are identical and equal to *σ* ^2^. A program is written in C++ language to employ the proposed RBFNN model.

**Figure 2:**
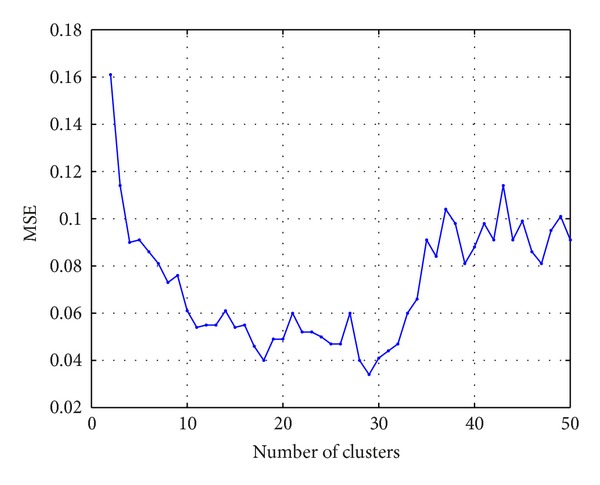
MSE versus number of clusters for proposed RBFNN.

The optimum number of hidden nodes for a RBFNN model should be carefully determined as it directly affects the performance of the network. In this study, in order to choose the optimum number of centers for the proposed network, several preliminary experiments are conducted by stepwise change of the number of centers from 2 to 50. For each case, an average mean square error (MSE) is calculated using the 10-fold cross-validation.  Figure 2 shows the MSE values with respect to the number of centers. Referring to Figure 2, the minimum MSE = 0.036 is achieved for 29 clusters and therefore the number of hidden nodes was set to 29.

After attaining the optimal RBFNN, the performance is compared to three different Pareto optimal three-layer MLP networks. In our study, MLP models were generated and implemented using the ANN module provided within the STATISTICA software (v 11.0) published by the Statsoft, Inc. MLP networks were constructed using the Automated Network Search (ANS) strategy for creating predictive models of STATISTICA. Best three MLP networks were retained by the ANS, trying different number of hidden units (1–30), different input/output activation functions (identity, logistic, tanh, and exponential) and different training algorithms such as the Gradient Descent, the Broyden-Fletcher-Goldfarh-Shanno (BFGS) (Quasi-Newton), the Conjugate Gradient Algorithm (CGA), or the Levenberg-Marquardt Algorithm using an error function of sum of squares. Moreover, a 10-fold cross-validation technique is selected to avoid overfitting and oscillation. The best three MLP networks which were determined using the ANS are summarized in Table 7. MLP-13 and MLP-23 employs the BFGS algorithm where the weights and biases are updated using the Hessian matrix performance index at the current values of the weights and biases. BFGS has high memory requirements due to storing the Hessian matrix. On the other hand, MLP-7 utilizes the CGA, which is a fast training algorithm for MLP networks that proceeds by a series of line searches through error space. In CGA, learning rate and momentum are calculated adaptively in each iteration. In the ANS module, the learning rate is calculated by the Golden Search rule while the Fletcher and Reeves formula [46] is used for momentum calculations.

Table 8 lists the mean of performance indicator results using the 10-fold cross-validation method for each network. Considering Table 8, it is noticeable that the mean classification *accuracy* of RBFNN (0.950) is better than any one of MLP networks (MLP-13 = 0.881, MLP-23 = 0.838, and MLP-7 = 0.800). Prediction capabilities based on AUC show that the proposed RBFNN outperforms all other MLP networks (RBFNN = 0.949, MLP-13 = 0.873, MLP-23 = 0.839, and MLP-7 = 0.793). The average *sensitivity* values for MLP networks are 0.896, 0.835, and 0.816 for MLP-13, MLP-23, and MLP-7, respectively. On the other hand, proposed RBFNN gives an average *sensitivity* of 0.953, which indicates that the RBFNN performs better on classifying cases having positive condition. Based on *specificity*, the RBFNN (94.8%) is superior to MLP-13 (86.8%), MLP-23 (84.0%), and MLP-7 (78.8%). *F-measure* and *Yuden Index* are the most widely used stand-alone performance indicators for classification studies. *F-measure* and *Yuden Index* values are 0.947 and 0.901 for the proposed RBFNN while 0.872 and 0.764 for MLP-13, 0.829, and 0.675 for MLP-23 and 0.783 and 0.604 for MLP-7, respectively. The mean PPV's are 0.849, 0.824, 0.753 and 0.942, while the mean NPV's are 0.909, 0.851, 0.843, and 0.958 for MLP-13, MLP-23, MLP-7, and RBFNN, respectively. These findings also show that a RBFNN performs better than MLP networks. In general, all models were good-fit models based on the *H*-*L* *statistics*  (*H*-*L* < 12.0).

In order to make precise and pairwise comparison between networks, two-tailed *t* tests are employed to show the statistical significance level of the difference of the mean of performance indicators for the RBFNN and MLP networks. Tables 9, 10, and 11 show the results of statistical tests. The mean, the standard deviation (SD), and the 95% confidence interval (CI) of each result are given in Tables 9–11. In the last column of Tables 9–11, a “+” sign denotes that the difference of performance indicator means is statistically significant at a 0.05 level, while a “–” sign indicates a difference which is not significant. The *t* test results clearly indicate that the difference between the proposed RBFNN network and MLP networks are statistically significant for all the indicators except the *H*-*L* statistic between MLP-23 and RBFNN. Therefore, it is evident that the proposed RBFNN is a better classifier for identifying the treatment type of femoral PAD's when compared to MLP networks.

## 6. Conclusion

In this work, an artificial intelligence model that determines the treatment type for femoral PAD is presented. The proposed model, which is based on the RBFNN framework, is compared to three Pareto optimal MLP networks using a repeated 10-fold cross-validation method for the reliability of results. The proposed RBFNN possesses superior performance than MLP networks in terms of performance measures such as AUC*, accuracy, sensitivity, specificity, positive predictive value, negative predictive value, F-score,* and *Yuden Index*. This work clearly indicates that RBFNN is a viable and powerful tool as a clinical decision support system for classifying the treatment options regarding femoral PADs. Future studies may cover using metaheuristic algorithms to determine optimal design parameters of RBFNNs such as the number and the location of centers or variances of clusters and as a result enhance the classification performance.

## Figures and Tables

**Table 1:** Features and their normalized values.

Feature	Comment
Age (years)	Divided by 100
Sex	Female = 0, male = 1
Fontaine stage	Stage I = 0, stage II-a = 0, stage II-b = 2, stage III = 3, stage IV = 4 (see Table 4)
Lesion type (TASC classification)	Type A = 0, type B = 1, type C = 3, type D = 4 (see Table 5)
Sensitivity to anesthesia	Low = 0, medium-high = 1
Distal bed	Absence = 0, presence = 1
Embolism (percent)	Divided by 100
LDL cholesterol level	Normal = 0, near/above normal = 1, BH = 2, high = 3, very high = 4 (see Table 3)
Smoking	Absence = 0, presence = 1
Exsmoker	Absence = 0, presence = 1
Hypertension	Absence = 0, presence = 1
Blood pressure	Normal = 0, pre-HTN = 1, stage I = 2, stage II = 3 (see Table 2)
Diabetes mellitus	Absence = 0, presence = 1
Other peripheral disease history	Absence = 0, presence = 1
Family history	Absence = 0, presence = 1
Current medical treatment	Absence = 0, presence = 1

Treatment decision	Medical treatment = −1, operation = 1

**Table 2:** Blood pressure level categories in adults.

Classification	Systolic pressure (mm Hg)	Diastolic pressure (mm Hg)
Normal	<120	<80
Prehypertension	120–139	80–89
Stage I	140–159	90–99
Stage II	>160	>100

**Table 3:** Cholesterol level categories in adults.

LDL cholesterol level (mg/dL)	LDL cholesterol category
<100	Optimal
100–129	Near optimal/above optimal
130–159	Borderline high
160–189	High
>190	Very high

**Table 4:** Fontaine stages [2].

Stages	Details
Stage I	Asymptomatic, incomplete blood vessel obstruction
Stage II-a	Claudication at a distance of greater than 200 meters
Stage II-b	Claudication distance of less than 200 meters
Stage III	Rest pain, mostly in the feet
Stage IV	Necrosis and/or gangrene of the limb

**Table 5:** TASC classification [3].

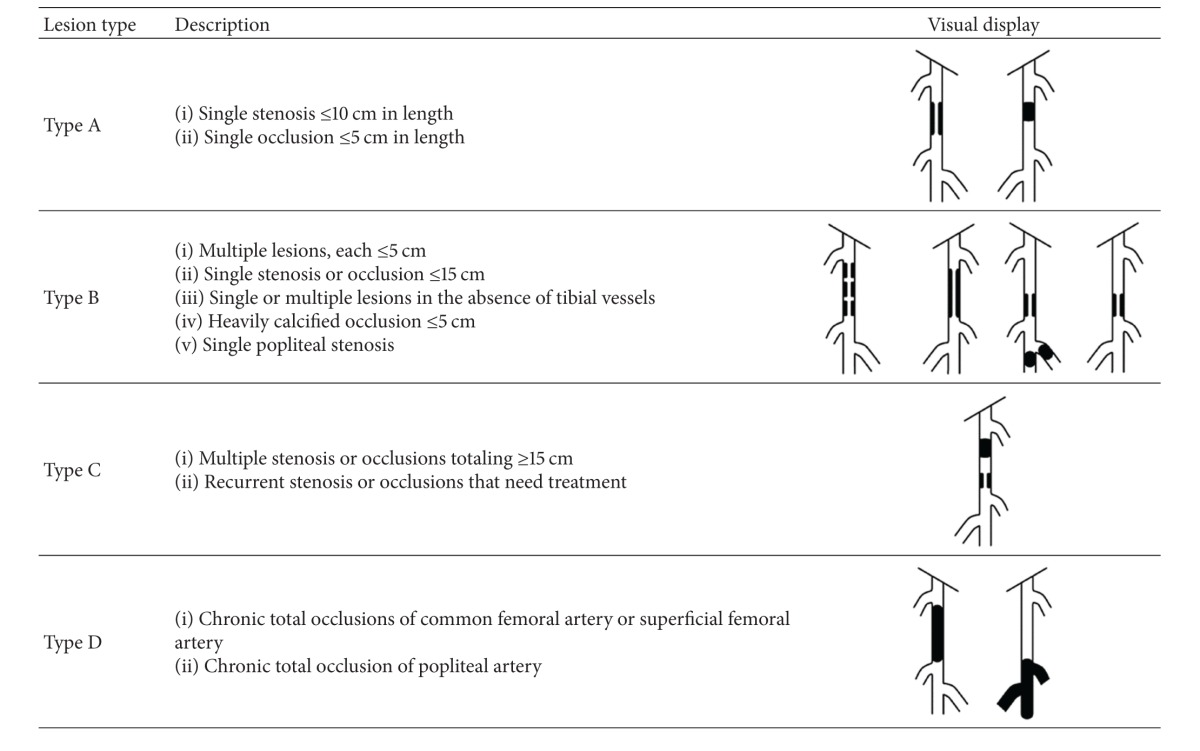

**Table 6:** Confusion matrix for binary classification.

Class/classified	As positive	As negative
Positive	tp	fn
Negative	fp	tn

**Table 7:** Selected MLP networks.

Network name	Training algorithm	Hidden activation function	Output activation function	Number of hidden units
MLP-13	BFGS	tanh	Logistic	13
MLP-23	BFGS	Identity	Logistic	23
MLP-7	CGA	Logistic	Identity	7

**Table 8:** Mean of performance indicators for MLP networks and RBFNN.

	MLP-13	MLP-23	MLP-7	RBFNN
AUC	0.873	0.839	0.793	0.949
Cutoff point	0.443	0.542	0.392	0.510
Accuracy	0.881	0.838	0.800	0.950
Sensitivity	0.896	0.835	0.816	0.953
Specificity	0.868	0.840	0.788	0.948
PPV	0.849	0.824	0.753	0.942
NPV	0.909	0.851	0.843	0.958
*F*-score	0.872	0.829	0.783	0.947
Yuden index	0.764	0.675	0.604	0.901
*H-L *	10.386	10.211	11.632	7.880

**Table 9:** Comparison of MLP-13 and RBFNN.

	MLP-13		RBFNN		Statistical significance
	Mean ± SD	95% CI	Mean ± SD	95% CI	
AUC	0.873 ± 0.018	0.862–0.885	0.949 ± 0.028	0.931–0.966	+
Cutoff	0.443 ± 0.010	0.437–0.449	0.510 ± 0.011	0.503–0.517	+
Accuracy	0.881 ± 0.016	0.871–0.891	0.950 ± 0.022	0.936–0.964	+
Sensitivity	0.896 ± 0.021	0.883–0.909	0.953 ± 0.015	0.944–0.963	+
Specificity	0.868 ± 0.018	0.857–0.879	0.948 ± 0.030	0.929–0.966	+
PPV	0.849 ± 0.023	0.835–0.864	0.942 ± 0.034	0.920–0.963	+
NPV	0.909 ± 0.019	0.897–0.921	0.958 ± 0.013	0.949–0.966	+
*F*-score	0.872 ± 0.018	0.861–0.883	0.947 ± 0.024	0.932–0.962	+
Yuden index	0.764 ± 0.033	0.744–0.785	0.901 ± 0.044	0.873–0.928	+
*H-L *	10.386 ± 2.125	9.069–11.703	7.880 ± 1.557	6.915–8.845	+

**Table 10:** Comparison of MLP-23 and RBFNN.

	MLP-23		RBFNN		Statistical significance
	Mean ± SD	95% CI	Mean ± SD	95% CI	
AUC	0.839 ± 0.018	0.828–0.850	0.949 ± 0.028	0.931–0.966	+
Cutoff	0.542 ± 0.016	0.532–0.552	0.510 ± 0.011	0.503–0.517	+
Accuracy	0.838 ± 0.017	0.827–0.848	0.950 ± 0.022	0.936–0.964	+
Sensitivity	0.835 ± 0.020	0.823–0.847	0.953 ± 0.015	0.944–0.963	+
Specificity	0.840 ± 0.018	0.829–0.851	0.948 ± 0.030	0.929–0.966	+
PPV	0.824 ± 0.021	0.811–0.836	0.942 ± 0.034	0.920–0.963	+
NPV	0.851 ± 0.019	0.839–0.862	0.958 ± 0.013	0.949–0.966	+
*F*-score	0.829 ± 0.018	0.818–0.840	0.947 ± 0.024	0.932–0.962	+
Yuden index	0.675 ± 0.034	0.654–0.697	0.901 ± 0.044	0.873–0.928	+
*H-L *	10.211 ± 3.409	8.098–12.324	7.880 ± 1.557	6.915–8.845	−

**Table 11:** Comparison of MLP-7 and RBFNN.

	MLP-7		RBFNN		Statistical significance
	Mean ± SD	95% CI	Mean ± SD	95% CI	
AUC	0.789 ± 0.019	0.778–0.801	0.949 ± 0.028	0.931–0.966	+
Cutoff	0.392 ± 0.009	0.386–0.398	0.510 ± 0.011	0.503–0.517	+
Accuracy	0.800 ± 0.020	0.787–0.812	0.950 ± 0.022	0.936–0.964	+
Sensitivity	0.823 ± 0.028	0.805–0.840	0.953 ± 0.015	0.944–0.963	+
Specificity	0.782 ± 0.017	0.772–0.792	0.948 ± 0.030	0.929–0.966	+
PPV	0.746 ± 0.021	0.733–0.759	0.942 ± 0.034	0.920–0.963	+
NPV	0.850 ± 0.025	0.834–0.865	0.958 ± 0.013	0.949–0.966	+
*F*-score	0.782 ± 0.022	0.769–0.796	0.947 ± 0.024	0.932–0.962	+
Yuden index	0.605 ± 0.041	0.579–0.630	0.901 ± 0.044	0.873–0.928	+
*H-L *	11.632 ± 2.169	10.288–12.976	7.880 ± 1.557	6.915–8.845	+
